# Acupuncture promotes mTOR-independent autophagic clearance of aggregation-prone proteins in mouse brain

**DOI:** 10.1038/srep19714

**Published:** 2016-01-21

**Authors:** Tian Tian, Yanhong Sun, Huangan Wu, Jian Pei, Jing Zhang, Yi Zhang, Lu Wang, Bin Li, Lihua Wang, Jiye Shi, Jun Hu, Chunhai Fan

**Affiliations:** 1 Division of Physical Biology & Bioimaging Center, Shanghai Synchrotron Radiation Facility, CAS Key Laboratory of Interfacial Physics and Technology, Shanghai Institute of Applied Physics, Chinese Academy of Sciences, Shanghai 201800, China; 2 Shanghai Institute of Acupuncture Moxibustion and Meridian, Shanghai University of Traditional Chinese Medicine, Shanghai 200030, China; 3 Department of Acupuncture and Moxibustion, Long Hua Hospital of Shanghai University of Traditional Chinese Medicine, Shanghai, 200032, China; 4 College of Acumox and Tuina, Shanghai University of Traditional Chinese Medicine, Shanghai 201203, China; 5 Kellogg College, University of Oxford, Banbury Road, Oxford, OX2 6PN, UK; 6 UCB Pharma, 208 Bath Road, Slough, SL1 3WE, UK

## Abstract

Acupuncture has historically been practiced to treat medical disorders by mechanically stimulating specific acupoints with fine needles. Despite its well-documented efficacy, its biological basis remains largely elusive. In this study, we found that mechanical stimulation at the acupoint of Yanglingquan (GB34) promoted the autophagic clearance of α-synuclein (α-syn), a well known aggregation-prone protein closely related to Parkinson’s disease (PD), in the substantia nigra par compacta (SNpc) of the brain in a PD mouse model. We found the protein clearance arose from the activation of the autophagy-lysosome pathway (ALP) in a mammalian target of rapamycin (mTOR)-independent approach. Further, we observed the recovery in the activity of dopaminergic neurons in SNpc, and improvement in the motor function at the behavior level of PD mice. Whereas acupuncture and rapamycin, a chemical mTOR inhibitor, show comparable α-syn clearance and therapeutic effects in the PD mouse model, the latter adopts a distinctly different, mTOR-dependent, autophagy induction process. Due to this fundamental difference, acupuncture may circumvent adverse effects of the rapamycin treatment. The newly discovered connection between acupuncture and autophagy not only provides a new route to understanding the molecular mechanism of acupuncture but also sheds new light on cost-effective and safe therapy of neurodegenerative diseases.

Aggregation-prone proteins, e.g. α-synuclein (α-syn), amyloid-β (aβ), Tau and polyglutamine-containing proteins (poly Q) are closely related to age-related neurodegeneration as seen in Parkinson’s disease (PD), Alzheimer’s disease (AD), Huntington’s disease (HD)^1^,^2^,^3^. For example, PD, one of the most common neurodegenerative diseases (NDDs) in the elderly, is closely related with α-syn aggregation in brain^2^,^3^. It has been well established that cytosolic and misfolded proteins are degraded via the ubiquitin proteasome system (UPS) and the autophagy-lysosome pathway (ALP) under physiological conditions^3^,^4^,^5^. Previous studies in mice demonstrated that impairment of basal autophagy perturbs protein degradation in the central nervous system and causes NDDs^6^. Hence, clearance of aggregation-prone proteins by enhancing the autophagic level holds great promise in therapeutic treatment for NDDs^3^,^5^.

Macroautophagy (hereinafter called autophagy) is a highly conservative biological degradation pathway that plays essential roles in homeostasis, development and survival^7^. As a specific inhibitor of the mammalian target of rapamycin (mTOR) signaling pathway, rapamycin has proven to be a potent inducer of autophagy and used for the treatment of certain diseases^3^,^8^. More specifically, it can attenuate toxicity of aggregate-prone proteins by activating ALP to clear protein aggregation in several types of NDDs^2^,^8^. However, rapamycin is known to have various adverse effects since it broadly affects many aspects of metabolism^8^. Hence, it is highly desirable to develop safer therapeutic approaches that specifically target downstream ALP in an mTOR-independent manner^3^.

Acupuncture is a treatment of traditional Chinese medicine (TCM) that mechanically stimulates discrete acupoints with fine needles. Acupuncture is practiced worldwide now, with accepted efficacies in treating many disorders and endorsement by the World Health Organization (WHO) and the US National Institutes of Health (NIH)^9^,^10^,^11^. However, the efficacy of acupuncture often raises considerable controversy in evidence-based medicine^12^, which largely arises from the lack of general mechanistic understanding of acupuncture. In this work, we aimed to study the positive roles of acupuncture for treating NDDs, which have been well documented for studies in experimental animals and patients^13^,^14^,^15^,^16^,^17^. Given that the nature of acupuncture is mechanical stimulation, and that autophagy is closely related with many physical factors, e.g. starvation, exercise and mechanical stress^18^,^19^, we were inspired to investigate the mechanism of acupuncture, the specificity of acupoints and PD treatment in a PD mouse model under the context of autophagic regulation.

## Results

### Acupuncture relieved the accumulation of α-synuclein in PD mice

**Figure 1:**
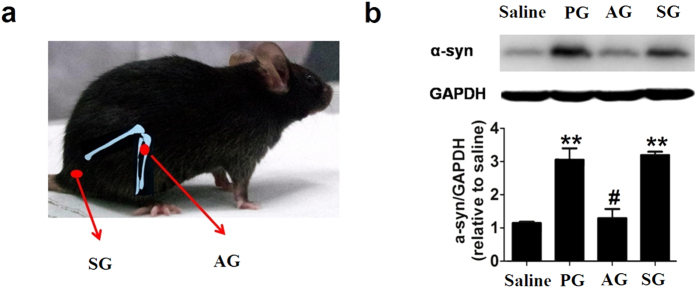
The acupoint position and α-synuclein expression. (**a**) AG: acupuncture at the acupoint of Yanglinquan (GB34); SG: sham acupuncture at the control point. (**b**) The expression of a-syn (top) and the optical density of α-syn (bottom) in the mouse SNpc in each group (7^th^ day after last MPTP injection). In all panels, *P < 0.05, **P < 0.01 compared with Saline group. ^#^*P* < 0.05, ^##^*P* < 0.01 compared with MPTP group. n = 8 per experimental group.

We used a PD mouse model by treating C57BL/6 mice with 1-methyl-4-phenyl-1,2,3,6-tetrahydropyridine (MPTP) at a dose of 30 mg/kg/d for five consecutive days (the PD mice group, PG), which is a well-established animal model with PD-like neuropathology^20^,^21^,^22^,^23^. We also found that the expression of α-syn was significantly increased in the substantia nigra par compacta (SNpc) of the PG mouse brain (Fig. 1). Concomitantly, we observed PD-like pathological changes including motor dysfunction and dopamine (DA) neuron impairment in SNpc of PG mice. Stimulation of Yanglingquan (GB34), which is located at the depression anterior and inferior to the fibular head^16^, is traditionally used for the motor function treatment in TCM^24^. Several previous studies have shown that acupuncture at GB34 can protect the dopaminergic neuron against MPTP^25^,^26^,^27^,^28^. Hence, we selected GB34 as the acupoint in this study. Acupuncture stimulation was performed once a day for 12 consecutive days at the acupoint GB34 in the acupuncture group (AG) or at a control point that is located at the lateral side (by 3 mm) of the tail in the sham acupuncture group (SG) (Fig. 1a)^25^,^26^,^27^,^28^. Significantly, we found that the expression level of α-syn in SNpc was decreased by ~56% in AG, which remained nearly constant in SG (Fig. 1b). This remarkable difference in treating different points reflects the site-specificity of acupoints and acupuncture, which is consistent with the acupuncture theory in TCM^29^,^30^.

### Acupuncture promoted the autophagic clearance of α-synuclein in an m-TOR-independent pathway

**Figure 2:**
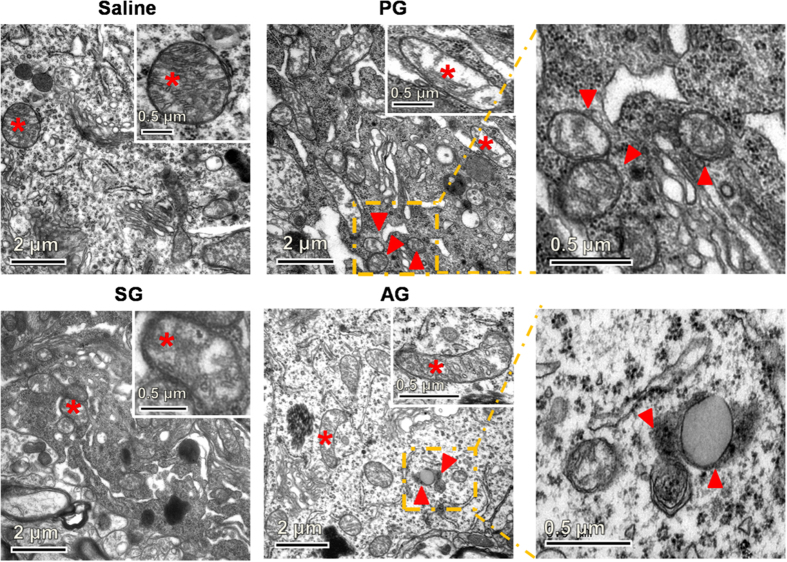
Transmission electron microscope (TEM) images for neuron cells in each group(7^th^ day after last MPTP injection). Arrowheads: autophagosome; asterisk: mitochondria, Scale bar: 2 μm; inset: magnified image for mitochondria(Scale bar: 0.5 μm).

Having established that acupuncture suppresses α-syn expression in SNpc, we explored the relationship between the observed α-syn clearance and autophagy. We first examined the sub-cellular structures in SNpc of mouse brain by using transmission electron microscopy (TEM). Mice in PG showed significant changes in the structure and distribution of mitochondria and lysosome as compared with those in the saline group (Fig. 2 and Supplementary Fig. 2). The mitochondria had a substantial loss of matrix density, swelling and cristae disruption, and the number of lysosomes significantly decreased. Notably, we found the appearance of abundant autophagosomes, which are characteristic of double-membrane structures containing intact or partly degraded cytoplasmic material^31^. The accumulation of autophagosomes along with the lysosomal impairment in PG mice are consistent with previous studies^23^, which suggests the dysfunction of the ALP in these mice with PD^27^. Remarkably, the acupuncture treatment at GB34 largely restored the structures of mitochondria and lysosome along with significant reduction of the autophagosome accumulation in AG (Fig. 2). In contrast, mice in SG did not show apparent effects on the structure of mitochondrion and lysosome (Fig. 2). Hence, acupuncture at the specific acupoint GB34 exhibits apparent autophagy induction in the SNpc of mice.

**Figure 3:**
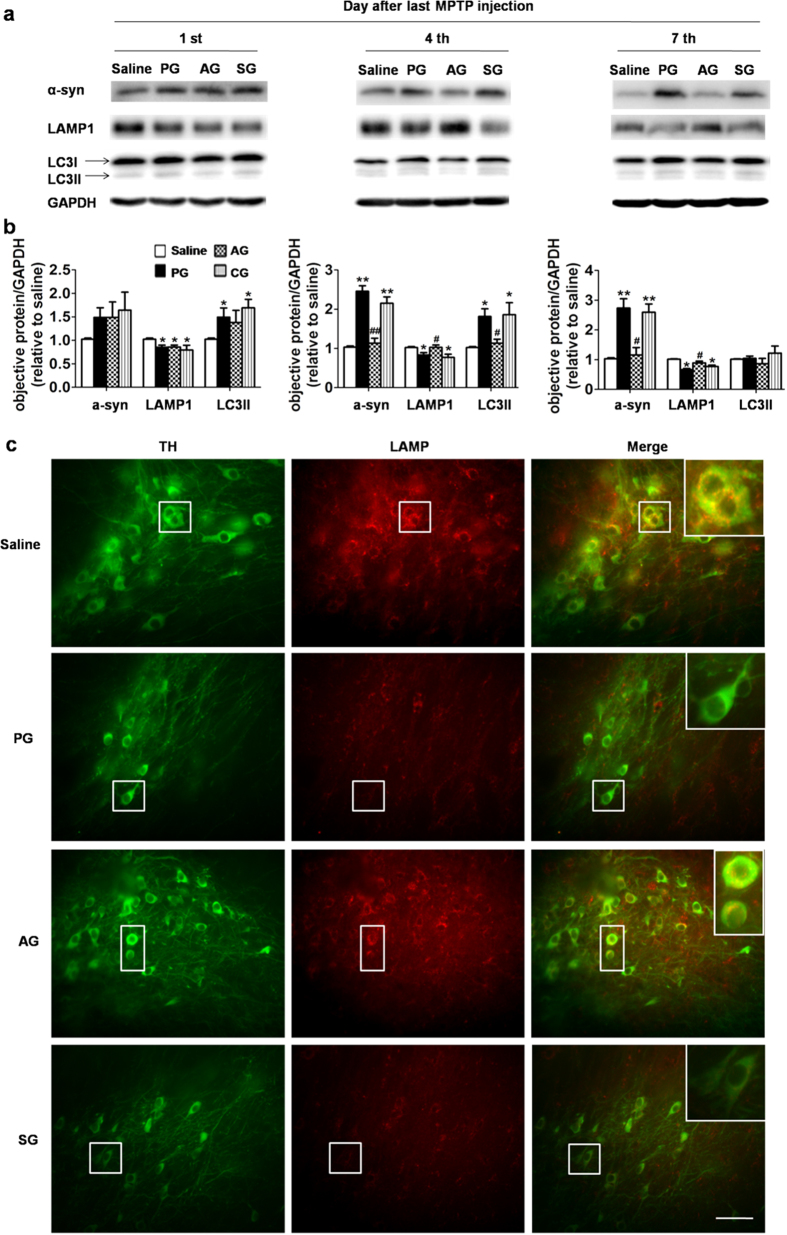
Acupuncture treatment at GB34 regulates the expression of α-syn and the autophagy-lysosome pathway in the substantia nigra of PD mice. (**a)** Immunoblot levels of α-syn, LAMP1 and LC3II in the mouse SNpc in AG for different days (1^st^, 4^th^, 7^th^ day after last MPTP injection). (**b**) the optical density of α-syn, LAMP1 and LC3II in SNpc. In all panels, *P < 0.05, **P < 0.01 compared with SG. ^#^*P* < 0.05, ^##^*P* < 0.01 compared with PG. n = 8 per experimental group. (**c**) The fluorescent images for TH (green) and LAMP2 (red) in SNpc of each group. n = 4–5 per experimental group. Scale bar: 100 μm.

We next analyzed the expression of ALP-related proteins to investigate the role of ALP in these processes. In ALP, microtubule-associated protein 1 light chain 3 II (LC3II) is a typical protein marker associated with the completed autophagosomes, and lysosome associated membrane protein 1 (LAMP1) is a lysosomal structural protein^23^,^31^. We first examined the expression levels of LC3II and LAMP1 in SNpc of PG mice. We found that LC3II was up-regulated, suggesting either the induction of autophagy or interruption of the fusion of autophagosome and lysosome^31^, and LAMP1 was down-regulated, suggesting the impairment of lysosome (Fig. 3a,b). Hence, it is the interruption of fusion of autophagosome and lysosome that leads to the accumulation of autophagosomes in SNpc of PG mice^31^. Significantly, compared with mice in PG, we observed ~37% decrease in the expression level of LC3II and ~20% in LAMP1 on the 4^th^ day in AG, along with over 50% clearance of α-syn in SNpc (Fig. 3a,b). These data suggest that acupuncture significantly rescues the ALP to enhance the clearance of autophagosomes and the degradation of α-syn. Double immunofluorescence examination was performed to locate lysosome with an anti-LAMP2 antibody and DA neurons with an anti-tyrosine hydroxylase (anti-TH) antibody, which further confirms the appearance of more lysosomes in SNpc DA neurons after the acupuncture treatment (Fig. 3c). We further note that the low expression of α-syn maintained till the 7^th^ day in AG, suggesting the sustainable effects of acupuncture. Also of note, mice in SG did not show apparent ALP restoration or significant α-syn clearance, which coincides well with the structural analysis in SNpc.

### Rapamycin enhanced α-syn clearance in an m-TOR-dependent pathway

**Figure 4:**
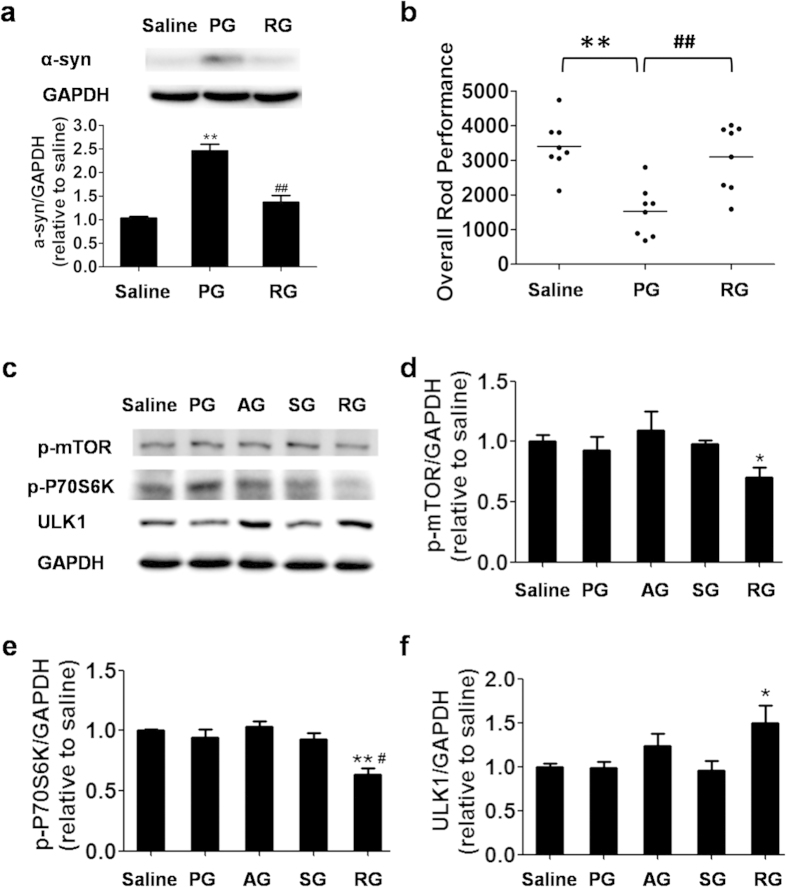
Rapamycin treatment decreases α-syn accumulation and restores the autophagy-lysosome pathway in the substantia nigra of PD mouse. (**a**) Immunoblot and the optical density of α-syn in the mouse SNpc in each group after rapamycin treatment for 12 days. (**b**) The overall rod performance (ORP) scores of mice in each group (n = 8). (**c**) Immunoblot levels of p-mTOR, p-P70S6K and ULK1 in the mouse SNpc in each group after acupuncture and rapamycin treatment. (**d–f**) The optical density of p-mTOR, p-P70S6K and ULK1 in the mouse SNpc in each group. In all panels, *P < 0.05, **P < 0.01 compared with SG. ^#^*P* < 0.05, ^##^*P* < 0.01 compared with PG.

Since LC3II and LAMP1 are both downstream ALP proteins, we further examined the acupuncture effects on the expression of upstream ALP proteins of p-mTOR, p-P70S6K and ULK1 (Fig. 4c–f). However, these upstream proteins were minimally affected by acupuncture, suggesting that its regulating effects on ALP are independent of mTOR^32^. We also compared acupuncture treatment with the treatment of an FDA-approved drug, rapamycin, which is a well known autophagic enhancer. The effects of rapamycin on downstream proteins (LC3II and LAMP1) in the rapamycin-treated group (RG) were generally similar to those of acupuncture (Fig. 4a and Supplementary Fig. 3c-e). Nevertheless, rapamycin could significantly modulate the expression of upstream ALP proteins including p-mTOR, p-P70S6K and ULK1 (Fig. 4c–f), suggesting that rapamycin not only restored the lysosomal level but also induced autophagy by inhibiting the m-TOR pathway.

### Acupuncture rescued the PD symptoms

**Figure 5:**
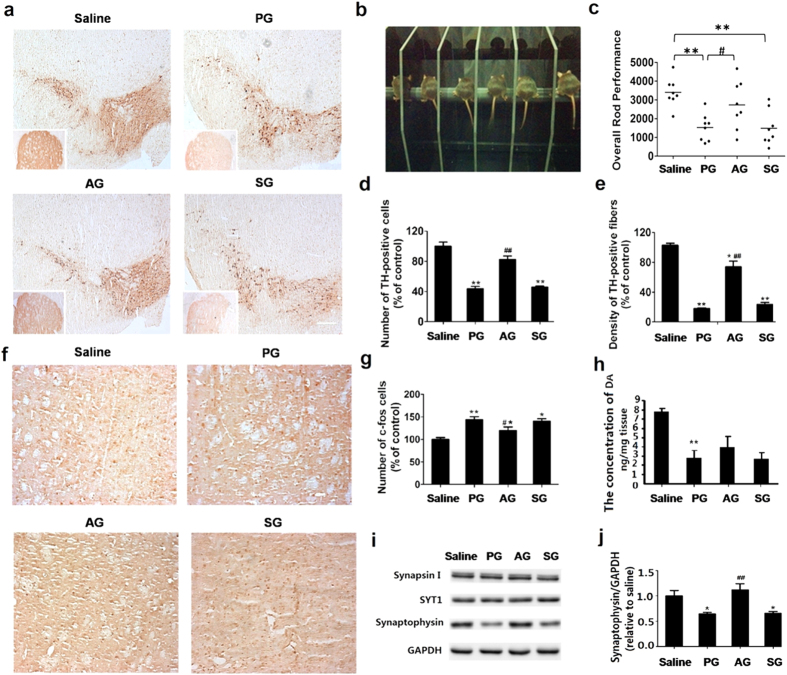
Acupuncture treatment at GB 34 improves exercise performance and protects dopamine neurons in PD mouse. (**a**) The TH-positive neurons in the SNpc and the TH-positive fibers in the striatum. Scale bar: 200 μm; inset: striatum. (**b**) Photograph for mice undergoing rota-rod testing. (**c**) The overall rod performance (ORP) scores for mice in each group (n = 8). (**d**) TH-positive neurons counts in the mouse SNpc in each group. (**e**) The optical density of TH-positive fibers in the mouse striatum in each group (n = 5). (**f**) Immunohistochemistry staining of c-fos in the striatum. (**g**) The number of c-fos positive cells in the striatum (n = 5). (**h**) The level of dopamine in the striatum. (**i**) Immunoblot for proteins involved in synaptic transmission in the striatum. (**j**) The optical density of synaptic Proteins. In all panels,*P < 0.01, **P < 0.01 compared with SG. ^#^*P* < 0.05, ^##^*P* < 0.01 compared with PG.

Having substantiated the acupuncture-induced autophagic clearance of α-syn, we next examined the therapeutic effect of acupuncture on the motor function, the number and the activity of DA neurons in mice of PG. PG mice were slow in movement with postural instability. In order to quantify their motor function, we measured the overall rod performance (ORP) score using a rota rod instrument^33^ (Fig. 5b). The ORP scores of mice in PG (1527.2 ± 238.6) were nearly half of those in the saline group (3401.6 ± 426.4). Remarkably, AG mice exhibited ~80% increase in the ORP scores (2728.1 ± 426.4), whereas SG ones did not show significant change (Fig. 5c). The observations on the behavior improvement are generally consistent with previous reported efficacies in acupuncture treatment for PD animals and patients^13^,^14^,^15^,^16^,^17^. We also note that PG mice showed sparse and dull hair coat, whereas that of AG mice was thick and shiny.

We further examined the number of TH-positive neurons and the level of DA, which reflect the number and the function of DA neurons, and the c-fos and synaptophysin proteins that reflect the activity of dopaminergic neuron. In PG mice, the number of TH-positive neurons in the SNpc and striatum respectively decreased by ~57% and ~78%, along with the reduction of DA concentration (PG: 2.72 ± 0.91 ng mg^−1^ tissue vs. saline: 7.72 ± 0.43 ng mg^−1^ tissue), whereas the c-fos expression increased by ~43% (Fig. 5a,d–h). After the acupuncture treatment at GB34, the number and function of DA neurons restored significantly in AG, with the density of TH-positive neurons in the SNpc and striatum increased by ~38% and ~61%, respectively (Fig. 5a,d,e). The DA concentration in AG was increased to 3.90 ± 1.22 ng mg^−1^ tissue(Fig. 5h). The synaptophysin protein expression in AG increased about by ~40% as compared with that in PG mice (Fig. 5i–j), which further confirmed that acupuncture rescued the loss of DA neurons in PG mice and improved the release of DA neurotransmitter. We also note that such a neuroprotective effect was not found in SG.

## Discussion

Although long-term TCM practice has accumulated evidence for the positive effects of acupuncture for treating many disorders. However, with few exceptions, the underlying molecular mechanism remains poorly established. In this study, we established the mechanistic connection between acupuncture and autophagy. As a type of mechanical stimulation, acupuncture can effectively restore the lysosomal level and act as a potent physical inducer for autophagic clearance of α-syn in PD mice. This molecular level change results in the recovery of DA neurons at organ level and the improvement of motor function at behavior level. Given that autophagy is a generic cellular regulation mechanism in response to physical (e.g. starvation and exercise) and chemical (e.g. rapamycin) stimuli, acupuncture, or more broadly mechanical stimulation, sheds new light on physically treating a wide range of diseases, increasing the health state and possibly prolongs life.

The effects of autophagic clearance of α-syn, protection of DA neuron and improvement in motor function in rapamycin-treated PD mice can be effectively replicated with acupuncture (Fig. 4a,b and Supplementary Fig. 3a,b). Rapamycin has been well known to prevent NDDs as an mTOR inhibitor^3^,^8^. However, since rapamycin influences both upstream and downstream ALP, and broadly affects cell growth and metabolism^8^,^34^, rapamycin-based treatments show adverse effects including dyslipidemias, anti-proliferative toxicity and renal dysfunction^35^, which has intrigued intense interest in developing new drugs independent of mTOR^3^ or finding new therapeutic targets^2^. In contrast, acupuncture treatments are mTOR-independent in nature, which specifically affects downstream ALP (Supplementary Fig. 4). This is a highly desirable feature for safe therapy of NDDs^3^. Hence, acupuncture holds great promise for treating NDDs by itself, or as a complement therapeutic tool to reduce the dose of rapamycin. We also expect that acupuncture might find applications in cancers and even longevity, in which rapamycin-based therapy shows positive effects^8^.

Cross-tissue coordination in response to stress has intrigued rapidly emerging interests^36^,^37^. Here, we demonstrated that stimulation at the GB34 acupoint on legs could remotely regulate ALP and protein expression in brain. In contrast, the sham acupuncture at an irrelevant site showed little effects at the molecular, organ and animal levels, which shows the site-specificity of acupuncture-induced effects. Although rapamycin can induce similar effects, it actually penetrates the blood brain barrier (BBB) due to its hydrophobicity. The remote regulation ability of acupuncture possibly arises from the transduction of mechanical signals via connective tissue^38^,^39^. Especially, Langevin and coworkers showed that stimulation at acupoints could produce amplificative mechanical signals^38^,^39^. When the acupuncture needle was inserted into the acupoint and rotated, mechanical signal was transmitted into cells with subsequent cellular response and downstream effects, such as cell secretion, modification of extracellular matrix, amplification and propagation of the signal, and modulation of afferent sensory. Nerve transmission might be another possible mechanism for the observed neuroprotective effects of acupuncture, as also observed in a recent electro-acupuncture study in treating system inflammation^11^. Although the mechanism of acupuncture’s remote regulation remains unclear, it opens new doors to circumvent BBB for targeted therapy in brain.

## Materials and Methods

### Chemicals and reagents

1-methyl-4-phenyl-1,2,3,6-tetrahydropyridine (MPTP)-HCl (Sigma-Aldrich, St. Louis, MO; M0896); Rapamycin (LC Laboratories, Woburn, MA; Cat# R-5000). Antibodies: anti-GAPDH (Sigma-Aldrich, St. Louis, MO; G9545, rabbit polyclonal, 1:5000 for western blot); anti-Tyrosine Hydroxylase (Millipore, Temecula, CA; Cat# MAB318, mouse monoclonal, 1:200 for immunofluorescence); anti-LC3 (Novus biological, Littleton, CO; NB100-2220, rabbit polyclonal, 1:1000 for western blot); anti-LAMP2 (abcam, Cambridge, MA; ab13524, rat monoclonal, 1:100 for immunofluorescence); anti-LAMP1 (Cell Signaling Technology, Boston, MA; Cat# 3243, rabbit polyclonal, 1:1000 for western blot); anti-ULK1 (abcam, Cambridge, MA; ab128859, rabbit monoclonal, 1:1000 for western blot); anti-Synaptophysin (Cell Signaling Technology, Boston, MA; Cat# 5461, rabbit polyclonal, 1:1000 for western blot); anti-SYT1 (Cell Signaling Technology, Boston, MA; Cat# 3347, rabbit polyclonal, 1:1000 for western blot); anti-Synapsin I (Millipore, Temecula, CA; Cat# AB1543, rabbit polyclonal, 1:1000 for western blot); anti-c-Fos (Santa Cruz Biotechnology, Santa Cruz, CA; Cat#: sc-52, rabbit polyclonal, 1:100 for immunohistochemistry); anti-α-synuclein (Santa Cruz Biotechnology, Santa Cruz, CA; Cat#: sc-7011-R, rabbit polyclonal, 1:500 for western blot); anti-phospho-mTOR (Cell Signaling Technology, Boston, MA; Cat# 5536, rabbit polyclonal, 1:1000 for western blot); anti-phospho-P70S6K (Cell Signaling Technology, Boston, MA; Cat# 9204, rabbit polyclonal, 1:1000 for western blot).

### Animals experiments

All animal experiments were approved by the ethics committee of Shanghai University of Traditional Chinese Medicine and conducted in strict accordance with the recommendations of the Guidelines for the Care and Use of Shanghai Laboratory Animal Center, Chinese Academy of Sciences. Male C57BL/6 mice (8~10-week-old, weighing 22–26 g) were obtained from Shanghai Laboratory Animal Center, Chinese Academy of Sciences and housed in the animal center of Shanghai University of Traditional Chinese Medicine. The mice were randomly divided into five groups, including the control group (Saline), the MPTP-treated PD mice group (PG), the acupuncture group (AG) stimulated at the acupoint GB34, the sham acupuncture group (SG) stimulated at the control point, and the rapamycin treatment group (RG), respectively (Supplementary Fig. 1). With the exception of the control group that was injected with saline, mice in all other groups received one intraperitoneal injection of 1-methyl-4-phenyl-1,2,3,6-tetrahydropyridine (MPTP)-HCl per day (30 mg/kg free base; Sigma-Aldrich, St. Louis, MO) for five consecutive days^21^.

Mice in RG received one intraperitoneal injection with rapamycin 30 min before the MPTP injection every day. Rapamycin was dissolved in saline adding 4% ethanol, 1% Tween 80, 5% Polyethylene glycol 400. The daily dose of rapamycin was of 10 mg/kg/d for the first two days, and then reduced to 5 mg/kg/d for the remaining ten days.

### Acupuncture treatment

Acupuncture stimulation was performed in AG two hours after the MPTP injection. Mice were lightly immobilized in the mouse holder, and then their legs were pulled out from holder to perform acupuncture treatment (Supplementary Fig. 1a). The acupuncture needles (10 mm in length, 0.19 mm in diameter, Huatuo Suzhou Medical Instruments Factory, Suzhou, China) were inserted to a depth of 3 mm at the GB34 in AG or the control point in SG. GB34 is located at the depression anterior and inferior to the fibular head^16^. Previous studies have proven that stimulation at GB34 showed better neuroprotective effects in the PD mouse model than that at houxi (SI3), shenmai (BL62) and zusanli (ST36)^26^. The control point was selected to be at 3 mm to the lateral side of the tail. The needles were rotated at a rate of two spins per sec for 15 sec according to previous studies^25^,^26^,^27^,^28^. They were then rotated for 15 sec every 5 min, and the acupuncture treatment lasted 10 min. During the acupuncture treatment, mice in other groups were also lightly immobilized in the mouse holder in the same way as those in AG.

### Behavioral test

We employed a Rota rod instrument (Chengdu TME Technology Co, Ltd.) to test the exercise performance of mice in each group. The overall rod performance (ORP) method was performed as described in the literature^33^. Before experiments, we first trained mice on the instrument at an accelerating speed and selected those could stay on the rod for 120 s at 20 rpm to conduct the experiments. On the 6^th^ day after the last MPTP injection, all mice were trained again on the instrument at an accelerating speed before testing. Then the testing was performed to record the time of mice staying on the rod at the successive rotational speeds (10, 15, 20, 25 and 30 rpm), with the maximum of 300 s at each speed. Finally, we calculated the overall rod performance (ORP) score for each mouse by the trapezoidal method^40^,^41^.

### Immunohistochemistry and immunofluorescence

On the 7^th^ day after the last MPTP injection, the mice were perfused transcardially with 4% paraformaldehyde in 0.1 M phosphate buffer. The removed substantia nigra and striatum were immersed overnight in 4% paraformaldehyde in 0.1 M phosphate buffer. For immunohistochemical staining, the brain tissues were cut at 5 μm thickness through the entire SNpc and striatum with the method of the paraffin sections. First, the paraffin sections were dewaxed, and then immersed in boiling solution (0.01 M sodium citrate buffer) 30 min for antigen retrieval. After antigen retrieval, the sections needed to be incubated with 3% H_2_O_2_ (dilution in methanol) for 10 min. Next, the sections were incubated with 6% bovine serum albumin (BSA) for 30 min at room temperature. After that, the sections were incubated with rabbit tyrosine hydroxylase antibody (TH, 1:500, Abcam) overnight at 4 °C. On the next day, the sections were incubated with biotinylated anti-rabbit IgG for 30 min at 37 °C, then incubated with horseradish peroxidase (HRP) for 10 min. The sections were incubated with 3,3’-diaminobenzidine (DAB, Histostain-Plus IHC Kit, MiaoTong) for 3 min. Finally, the sections were dehydrated and covered, and imaged by a bright-field microscope. We selected five consecutive sections at the same anatomical position from one sample to count the TH-positive cells in the SNpc, and used the same method to analyze the DA neurons in the striatum. TH-positive cells in the SNpc were counted by using the Image J software. Optical densities of dopamine neurons in the striatum were also analyzed with the same software.

For immunofluorescence staining, brain tissues were removed and post-fixed in 4% paraformaldehyde for 2 h, then the tissues were immersed in 30% sucrose overnight at 4 °C. The tissues were sectioned at 30 μm using a vibratome (Leica), the sections through the entire substantia nigra. The free-floating sections were incubated in 0.25% Triton X-100 in PBS for 15 min. After being washed in PBST for 30 min, the sections were incubated with 6% BSA for 30 min at room temperature. Next, the sections were co-incubated overnight at 4 °C with anti-tyrosine hydroxylase monoclonal antibody (TH, mouse, 1:200, Millipore) and anti- lysosome associated membrane protein 2 monoclonal antibody (LAMP2, rat, 1:200, Abcam). On the next day, the sections were co-incubated with Alexa 488 and 594 after washing for 30 min. Finally, the sections were covered using the anti-fade reagent (Invitrogen), and then examined with the confocal microscope (Leica).

### Western Blotting

The mice were sacrificed on the days of 1, 4 and 7 after the last MPTP injection. The brain tissues were removed and the SNpc were quickly dissected on the ice. The tissues were homogenized in protein lysis buffer which composed by 150 mM NaCl, 5 mM EDTA, 50 mM Tris-HCl (pH 7.0), 1% Nonidet P-40, 1% SDS and Mini mixture protease inhibitors (Roche Diagnostics), and the protein concentration was detected using the BCA protein assay kit (Thermo Scientific). The samples were then boiled with loading buffer at 95 °C for 5 min, subpackaged into EP tube, and stored at −80 °C. We employed 4–12% SDS/PAGE gel to separate the protein samples. PVDF membranes were first blocking for 1 h in 5% nonfat milk (dry milk dissolved into 0.1 M PBS with 0.1% Tween-20), and then incubated overnight at 4 °C with a primary antibody (α-syn, rabbit, 1:500, santa cruz; LC3, rabbit, 1:1000, Novus; LAMP-1, rabbit, 1:1000, Abcam). On the next day, membranes were washed with PBST for 30 min, then incubated with an HRP-conjugated anti-rabbit antibody (1:10000, Sigma) at room temperature for 1 h. After being washed for 30 min, they were incubated with an ECL kit (Millipore) for 30 s. The signal was detected using a Gel & Blot Imaging system (Syngene). The band intensity of the proteins was quantified using the Quantity One software (Bio-Rad).

### Transmission electron microscopy

The mice were perfused transcardially with 4% paraformaldehyde and 1% glutaraldehyde in 0.1 M phosphate buffer. The removed substantia nigra was immersed in 2.5% glutaraldehyde for 2 h. The sections were cut at 100 μm using a vibratome (Leica), and then fixed in 1% osmium teroxide for 1 h. The temperature was controlled at 4 °C. The sections were dehydrated, and embedded in epoxy resin, then cut into 70 nm. Finally, the sections were stained with 5% uranyl acetate, then imaged under TEM.

### Statistical Analysis

The Graph Pad Prism software (v5.0) was employed for statistical analysis. All data were presented as mean ± standard error of the mean (SEM). Statistics among each experimental group was analyzed using a Student t test or a one-way ANOVA followed by Bonferroni’s post hoc test. In all analyses, statistical significance was considered at P < 0.05.

## Additional Information

**How to cite this article**: Tian, T. *et al.* Acupuncture promotes mTOR-independent autophagic clearance of aggregation-prone proteins in mouse brain. *Sci. Rep.* **6**, 19714; doi: 10.1038/srep19714 (2016).

## Supplementary Material

Supplementary Information

## References

[b1] Ross C. A. & Poirier M. A. Protein aggregation and neurodegenerative disease. Nat. Med. 10, S10–S17 (2004). 15272267 10.1038/nm1066

[b2] Decressac M. *et al.* TFEB-mediated autophagy rescues midbrain dopamine neurons from alpha-synuclein toxicity. Proc. Natl. Acad. Sci. USA 110, E1817–E1826 (2013). 23610405 10.1073/pnas.1305623110 PMC3651458

[b3] Bove J., Martinez-Vicente M. & Vila M. Fighting neurodegeneration with rapamycin: mechanistic insights. Nat. Rev. Neurosci. 12, 437–452 (2011). 21772323 10.1038/nrn3068

[b4] Rubinsztein D. C. The roles of intracellular protein-degradation pathways in neurodegeneration. Nature 443, 780–786 (2006). 17051204 10.1038/nature05291

[b5] Wong E. & Cuervo A. M. Autophagy gone awry in neurodegenerative diseases. Nat. Neurosci. 13, 805–811 (2010). 20581817 10.1038/nn.2575 PMC4038747

[b6] Hara T. *et al.* Suppression of basal autophagy in neural cells causes neurodegenerative disease in mice. Nature 441, 885–889 (2006). 16625204 10.1038/nature04724

[b7] Levine B. & Kroemer G. Autophagy in the pathogenesis of disease. Cell 132, 27–42 (2008). 18191218 10.1016/j.cell.2007.12.018 PMC2696814

[b8] Li J., Kim S. G. & Blenis J. Rapamycin: One Drug, Many Effects. Cell Metabolism 19, 373–379 (2014). 24508508 10.1016/j.cmet.2014.01.001 PMC3972801

[b9] Bonafede M., Dick A., Noyes K., Klein J. D. & Brown T. The effect of acupuncture utilization on healthcare utilization. Med. Care 46, 41–48 (2008). 18162854 10.1097/MLR.0b013e3181589b7d

[b10] Goldman N. *et al.* Adenosine A1 receptors mediate local anti-nociceptive effects of acupuncture. Nat. Neurosci. 13, 883–888 (2010). 20512135 10.1038/nn.2562 PMC3467968

[b11] Torres-Rosas R. *et al.* Dopamine mediates vagal modulation of the immune system by electroacupuncture. Nat. Med. 20, 291–295 (2014). 24562381 10.1038/nm.3479 PMC3949155

[b12] Hinman R. S. *et al.* Acupuncture for Chronic Knee Pain A Randomized Clinical Trial. Jama-Journal of the American Medical Association 312, 1313–1322 (2014). 10.1001/jama.2014.12660 25268438

[b13] Cristian A., Katz M., Cutrone E. & Walker R. H. Evaluation of acupuncture in the treatment of Parkinson’s disease: A double-blind pilot study. Mov. Disord. 20, 1185–1188 (2005). 15884039 10.1002/mds.20503

[b14] Chae Y. *et al.* Parsing Brain Activity Associated with Acupuncture Treatment in Parkinson’s Diseases. Mov. Disord. 24, 1794–1802 (2009). 19533753 10.1002/mds.22673

[b15] Yu Y. P. *et al.* Acupuncture inhibits oxidative stress and rotational behavior in 6-hydroxydopamine lesioned rat. Brain Res. 1336, 58–65 (2010). 20399757 10.1016/j.brainres.2010.04.020

[b16] Kim S.-N. *et al.* Acupuncture Enhances the Synaptic Dopamine Availability to Improve Motor Function in a Mouse Model of Parkinson’s Disease. PLoS One 6, e27566 (2011). 22132113 10.1371/journal.pone.0027566 PMC3222639

[b17] Yeo S. *et al.* Acupuncture Stimulation on GB34 Activates Neural Responses Associated with Parkinson’s Disease. CNS Neurosci. Therap. 18, 781–790 (2012). 22943145 10.1111/j.1755-5949.2012.00363.x PMC6493472

[b18] He C. C. *et al.* Exercise-induced BCL2-regulated autophagy is required for muscle glucose homeostasis. Nature 481, 511–515 (2012). 22258505 10.1038/nature10758 PMC3518436

[b19] King J. S., Veltman D. M. & Insall R. H. The induction of autophagy by mechanical stress. Autophagy 7, 1490–1499 (2011). 22024750 10.4161/auto.7.12.17924 PMC3327616

[b20] Langston J. W. *et al.* Evidence of active nerve cell degeneration in the substantia nigra of humans years after 1-methyl-4-phenyl-1, 2, 3, 6-tetrahydropyridine exposure. Ann. Neurol. 46, 598–605 (1999). 10514096 10.1002/1531-8249(199910)46:4<598::aid-ana7>3.0.co;2-f

[b21] Jackson-Lewis V. & Przedborski S. Protocol for the MPTP mouse model of Parkinson’s disease. Nat. Protoc. 2, 141–151 (2007). 17401348 10.1038/nprot.2006.342

[b22] Vila M. *et al.* a-Synuclein Up-Regulation in Substantia Nigra Dopaminergic Neurons Following Administration of the Parkinsonian Toxin MPTP. J Neurochem 74, 721–729 (2000). 10646524 10.1046/j.1471-4159.2000.740721.x

[b23] Dehay B. *et al.* Pathogenic Lysosomal Depletion in Parkinson’s Disease. J. Neurosci. 30, 12535–12544 (2010). 20844148 10.1523/JNEUROSCI.1920-10.2010 PMC6633458

[b24] Yeo S. *et al.* Acupuncture on GB34 activates the precentral gyrus and prefrontal cortex in Parkinson’s disease. BMC Complement. Altern. Med. 14, 336 (2014). 25220656 10.1186/1472-6882-14-336 PMC4175221

[b25] Kang J. M. *et al.* Acupuncture inhibits microglial activation and inflammatory events in the MPTP-induced mouse model. Brain Res 1131, 211–219 (2007) 17173870 10.1016/j.brainres.2006.10.089

[b26] Jeon S. *et al.* Proteomic analysis of the neuroprotective mechanisms of acupuncture treatment in a Parkinson’s disease mouse model. Proteomics 8, 4822–4832 (2008). 18942673 10.1002/pmic.200700955

[b27] Choi Y. G., Park J. H. & Lim S. Acupuncture inhibits ferric iron deposition and ferritin-heavy chain reduction in an MPTP-induced parkinsonism model. Neurosci. Lett. 450, 92–96 (2009). 19056464 10.1016/j.neulet.2008.11.049

[b28] Hong M. S. *et al.* Gene expression profile of acupuncture treatment in 1-methyl-4-phenyl-1,2,3,6-tetrahydropyridine-induced Parkinson’s disease model. Neurol. Res. 32, S74–S78 (2010). 10.1179/016164109X12537002794165 20034450

[b29] Ramsay D. J. *et al.* Acupuncture. Jama-Journal of the American Medical Association 280, 1518–1524 (1998).

[b30] Kaptchuk T. J. Acupuncture: Theory, efficacy, and practice. Ann. Intern. Med. 136, 374–383 (2002). 11874310 10.7326/0003-4819-136-5-200203050-00010

[b31] Klionsky D. J. *et al.* Guidelines for the use and interpretation of assays for monitoring autophagy. Autophagy 8, 445–544 (2012). 22966490 10.4161/auto.19496 PMC3404883

[b32] Fleming A., Noda T., Yoshimori T. & Rubinsztein D. C. Chemical modulators of autophagy as biological probes and potential therapeutics. Nat. Chem. Biol. 7, 9–17 (2011). 21164513 10.1038/nchembio.500

[b33] Rozas G., Lopez-Martin E., Guerra M. J. & Labandeira-Garcia J. L. The overall rod performance test in the MPTP-treated-mouse model of Parkinsonism. J. Neurosci. Methods 83, 165–175 (1998). 9765130 10.1016/s0165-0270(98)00078-8

[b34] Laplante M. & Sabatini D. M. mTOR Signaling in Growth Control and Disease. Cell 149, 274–293 (2012). 22500797 10.1016/j.cell.2012.03.017 PMC3331679

[b35] Kahan B. Toxicity spectrum of inhibitors of mammalian target of rapamycin in organ transplantation: etiology, pathogenesis and treatment. Expert Opin. Drug Saf. 10, 727–749 (2011). 21557712 10.1517/14740338.2011.579898

[b36] Taylor R. C., Berendzen K. M. & Dillin A. Systemic stress signalling: understanding the cell non-autonomous control of proteostasis. Nat. Rev. Mol. Cell Biol. 15, 211–217 (2014). 24556842 10.1038/nrm3752 PMC5922984

[b37] Ulgherait M., Rana A., Rera M., Graniel J. & Walker D. W. AMPK Modulates Tissue and Organismal Aging in a Non-Cell-Autonomous Manner. Cell Rep. 8, 1767–1780 (2014). 25199830 10.1016/j.celrep.2014.08.006 PMC4177313

[b38] Langevin H. M., Churchill D. L. & Cipolla M. J. Mechanical signaling through connective tissue: a mechanism for the therapeutic effect of acupuncture. FASEB J. 15, 2275–2282 (2001). 11641255 10.1096/fj.01-0015hyp

[b39] Langevin H. M. & Yandow J. A. Relationship of acupuncture points and meridians to connective tissue planes. The Anatomical record 269, 257–265 (2002). 12467083 10.1002/ar.10185

[b40] Rozas G., Guerra M. J. & Labandeira-Garcia J. L. An automated rotarod method for quantitative drug-free evaluation of overall motor deficits in rat models of parkinsonism. Brain Research Protocols 2, 75–84 (1997). 9438075 10.1016/s1385-299x(97)00034-2

[b41] Tian T. *et al.* Synchrotron radiation X-ray fluorescence analysis of Fe, Zn and Cu in mice brain associated with Parkinson’s disease. Nucl. Sci. Tech. 26, 030506 (2015).

