# Supplementary material for: Acupuncture promotes mTOR-independent autophagic clearance of aggregation-prone proteins in mouse brain
Source: Sci Rep. 2016 Jan 21;6:19714. doi: 10.1038/srep19714 (PMC4726430; doi:10.1038/srep19714)
Supplement: Supplementary Information [file srep19714-s1.doc]

**Supplementary Information**

**Acupuncture promotes mTOR-independent autophagic clearance of aggregation-prone proteins in mice brain**

Tian Tian^1^, Yanhong Sun^1^, Huangan Wu^2^, Jian Pei ^3^, Jing Zhang^4^, Yi Zhang^1^, Lu Wang^1^, Bin Li^1^, Lihua Wang^1^, Jiye Shi^5^, Jun Hu^1^, Chunhai Fan^1^*

**
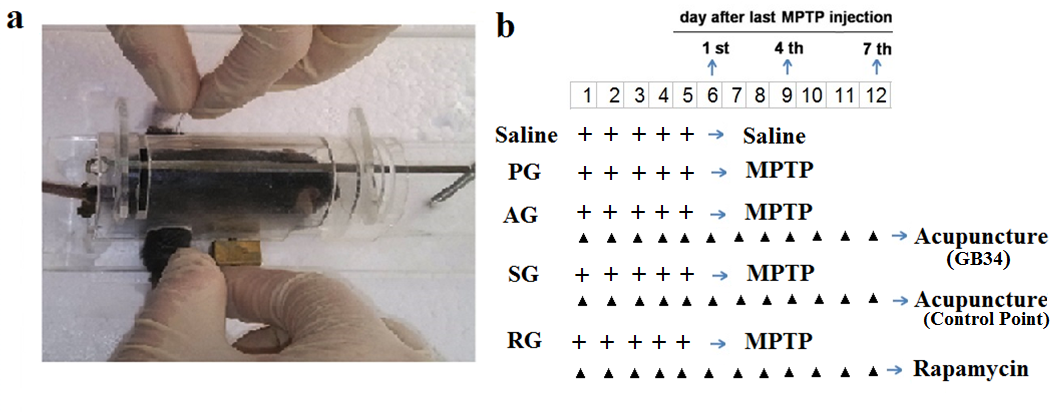
**

Supplementary Figure 1. Acupuncture treatment and experimental procedure (a) Acupuncture treatment at GB34 (b) the experimental procedure.


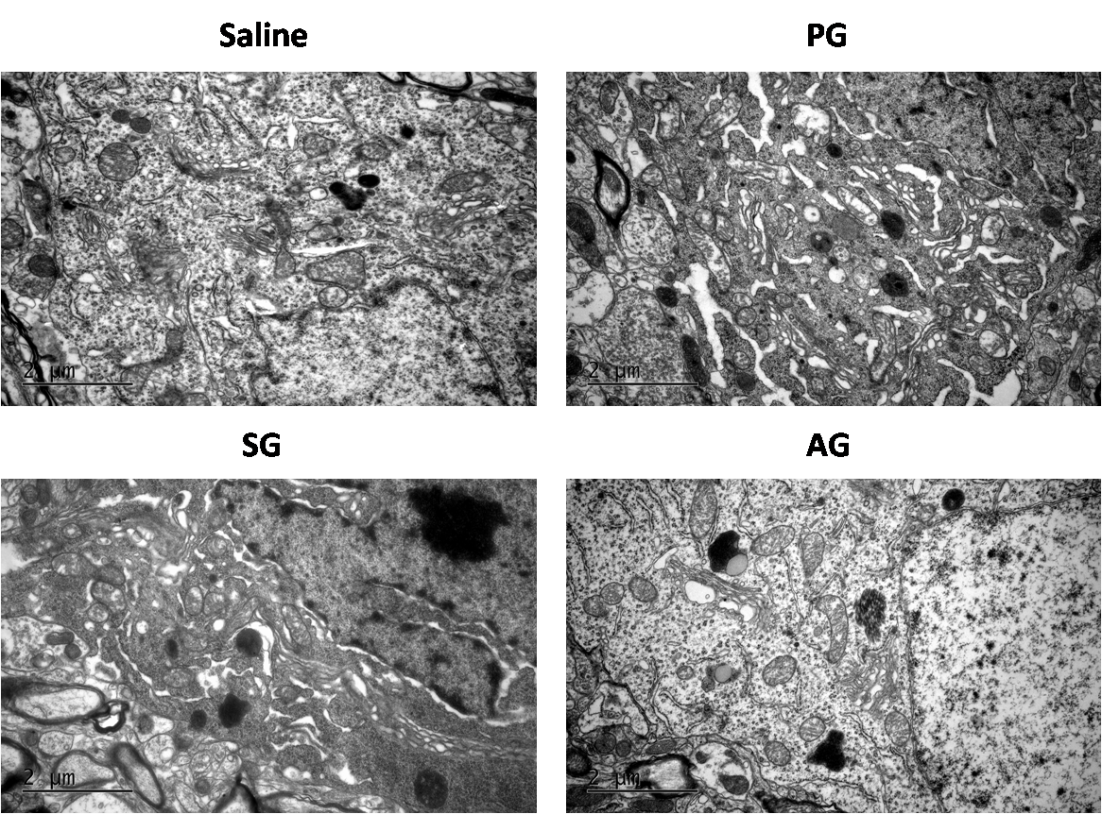


Supplementary Figure 2. the large area TEM images for neuron cells in each group at lower magnification. (7^th^ day after last MPTP injection, Scale bar: 2μm)

**
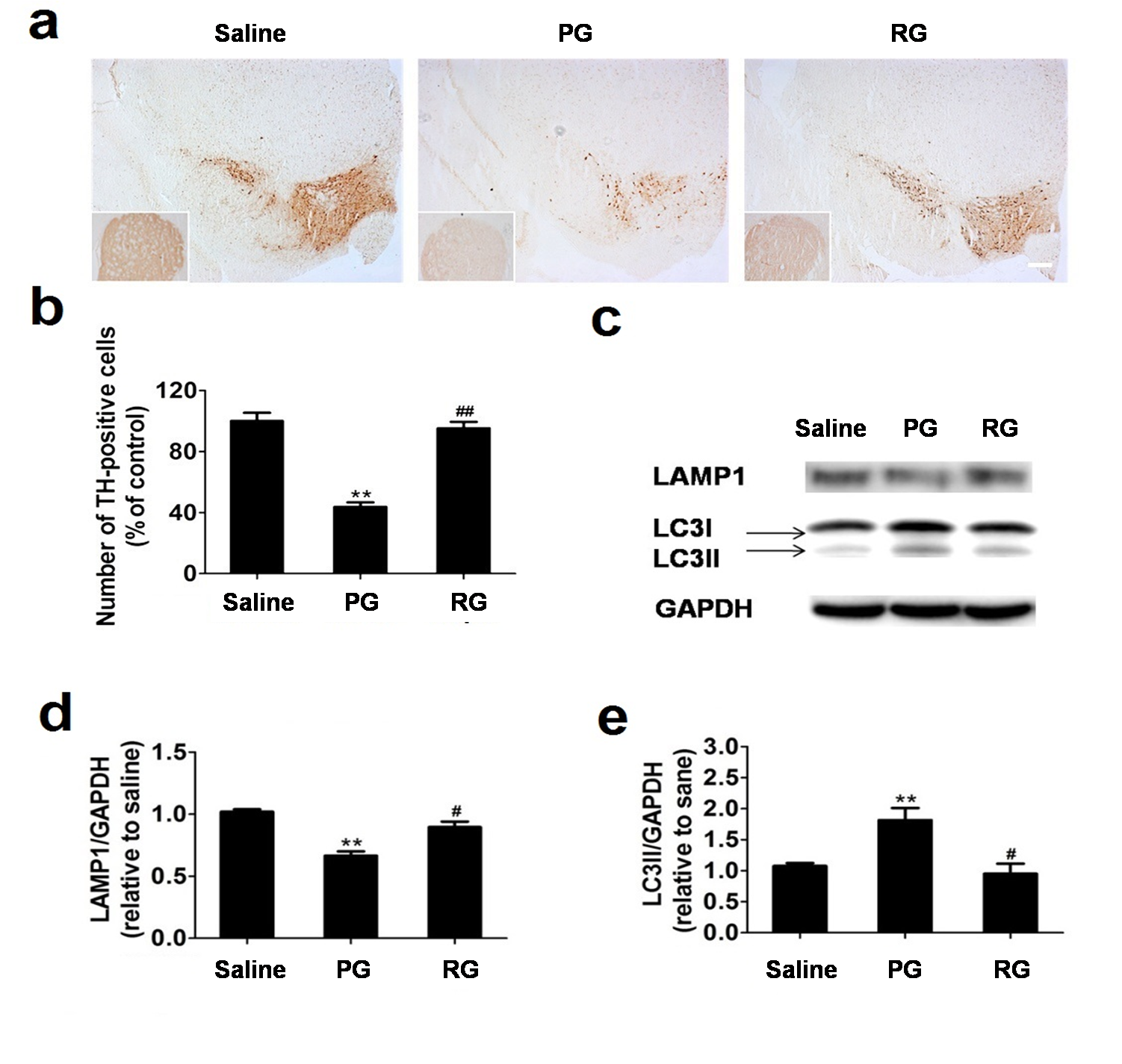
**

Supplementary Figure 3. Rapamycin protected neurons against MPTP in mice. (a) The TH-positive neurons in the substantia nigra. Scale bar, 200 μm. (b) The TH-positive neurons counts in the substantia nigra in SG and PG (n=5). (c) LAMP1 and LC3II immunoblot levels in SNpc of each group.（d-e） the optical density of LAMP1 and LC3II. In all panels, ***P<*0.01 compared with SG. #*P*<0.05, ## *P*<0.01 compared with PG.


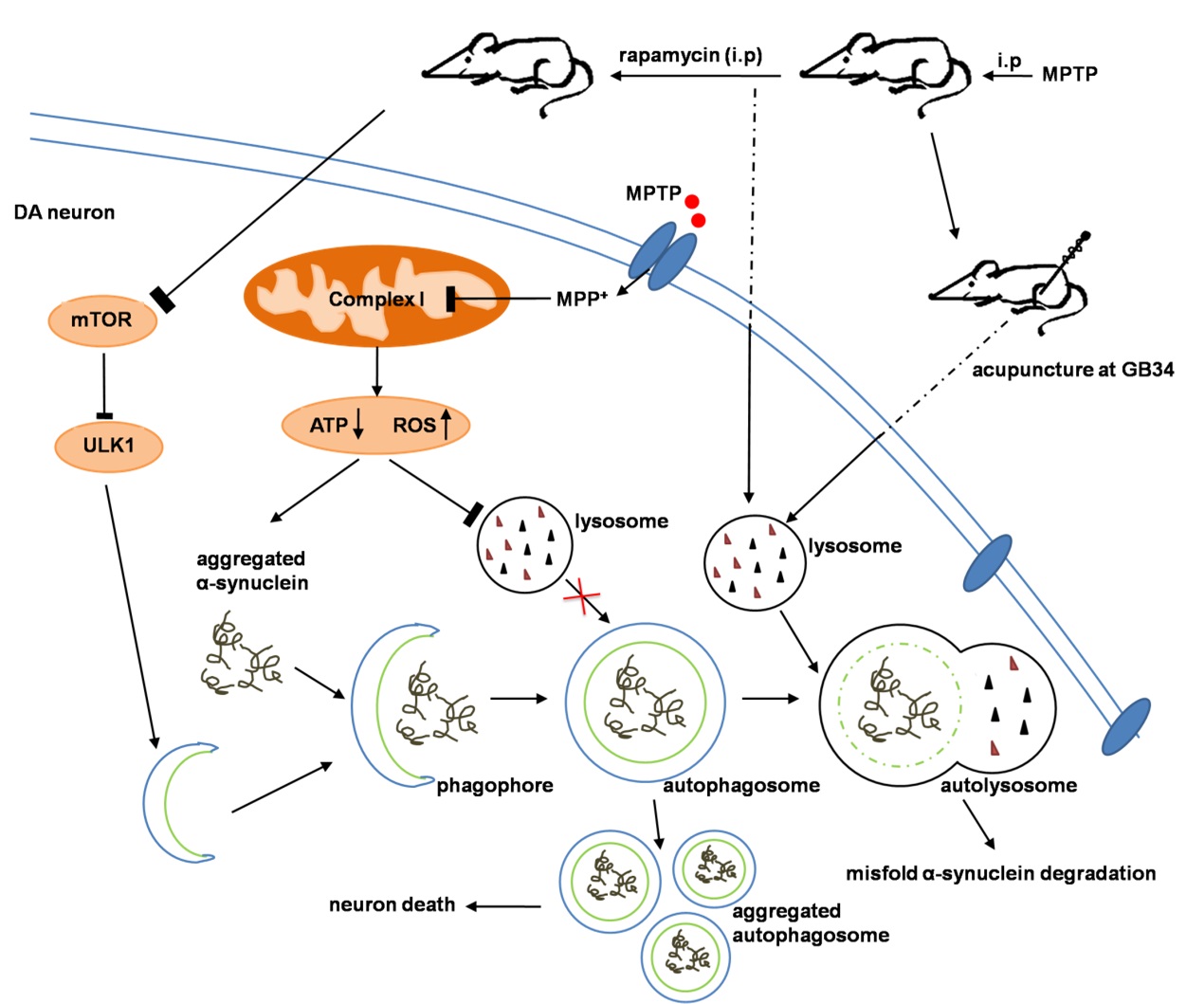


Supplementary Figure 4 . Acupuncture promotes autophagic clearance of aggregation-prone proteins in an m-TOR-independent pathway.
